# Systolic versus diastolic myocardial blood flow in patients with suspected coronary artery disease - a cardiovascular magnetic resonance study

**DOI:** 10.1186/1532-429X-14-S1-P17

**Published:** 2012-02-01

**Authors:** Manish Motwani, Timothy Fairbairn, Abdulghani M Larghat, Adam N Mather, John D Biglands, Aleksandra Radjenovic, John P Greenwood, Sven Plein

**Affiliations:** 1 Multidisciplinary Cardiovascular Research Centre & Leeds Institute of Genetics, Health and Therapeutics, University of Leeds, Leeds, UK; 2 Division of Medical Physics, University of Leeds, Leeds, UK; 3 NIHR Leeds Musculoskeletal Biomedical Research Unit, University of Leeds, Leeds, UK

## Summary

This study has shown that in patients with suspected and confirmed CAD, estimates of MBF by perfusion-CMR are significantly higher in diastole than systole during maximal hyperemic stress.

## Background

Differences in myocardial blood flow (MBF) between systole and diastole have been reported in healthy volunteers but the impact of cardiac phase on detecting coronary artery disease (CAD) is unknown [1]. This study aimed to compare MBF estimates from cardiovascular magnetic resonance (perfusion-CMR) imaging in systole and diastole in patients with suspected CAD and determine if either phase has greater diagnostic accuracy.

## Methods

Following invasive coronary angiography, 40 patients (68% men, 64 ± 8 yrs) underwent stress perfusion-CMR (1.5T Philips) acquired at mid-systole and end-diastole simultaneously [1]. Based on angiographic stenosis >70%, patients were grouped as having ‘CAD’ or ‘no CAD’. In patients with CAD, myocardial segments were classified as ‘stenosis-dependent’ (downstream of a significant stenosis) or ‘remote’. For each segment, MBF (Fermi-constrained deconvolution) and myocardial perfusion reserve (MPR) were calculated. The diagnostic accuracy of each phase was determined with receiver operator characteristic analysis.

## Results

**Figure 1:**
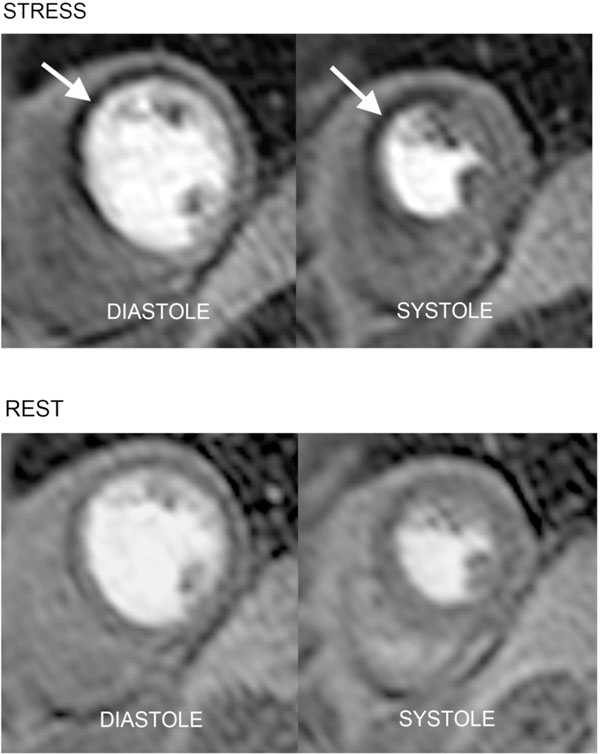
Example perfusion-CMR images with acquisition in diastole and systole. This patient had a subtotal occlusion of the left anterior descending artery. Corresponding stress perfusion defects (white arrows) are seen in the anterior, anteroseptal and inferoseptal segments of a mid-ventricular slice acquired in both diastole and systole.

**Figure 2:**
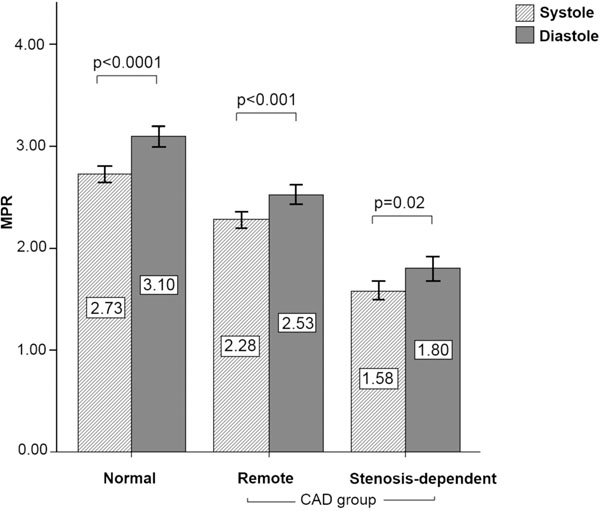
Comparison of MPR between systole and diastole. Segmental MPR (mean ± SEM) is shown in diastole and systole for normal segments, remote CAD segments and stenosis-dependent CAD segments.

Twenty-one patients (53%) had CAD. A typical example of a patient with ischemia is shown in Figure 1. Resting MBF was similar in the two cardiac phases for both normal and CAD patients (all p values > 0.05). MBF at stress was greater in diastole than systole in normal, remote and stenosis-dependent segments (3.75 ± 1.5 vs. 3.15 ± 1.1 ml/g/min; 2.75 ± 1.20 vs. 2.38 ± 0.99 ml/g/min; 2.49 ± 1.07 vs. 2.23 ± 0.90 ml/g/min; all p values < 0.01). MPR was also greater in diastole than systole in all three segment groups (all p values <0.05) (Figure 2). On receiver operator characteristic analysis, the optimal MPR cut-off for the detection of CAD was 1.95 for systole and 2.04 for diastole (area under curve 0.82 vs. 0.79; p=0.30).

## Conclusions

Estimates of stress MBF and MPR by perfusion-CMR in this study were greater in diastole than systole in normal and CAD patients. Although the diagnostic accuracy of both phases was similar, the MPR cut-off values were different. These observations are particularly important in the emerging field of 3D perfusion-CMR where the acquisition phase may be specifically chosen. Different estimates of MBF and different MPR cut-off values between phases mean a universal standard needs to be agreed for 3D acquisitions.

## Funding

S.P is funded by a British Heart Foundation fellowship (FS/10/62/28409).

S.P and J.P.G received an unrestricted educational research grant from Philips Healthcare.
